# "What kills neurons in neurodegenerative diseases?", a review series in an open access journal

**DOI:** 10.1186/1750-1326-4-7

**Published:** 2009-02-04

**Authors:** Todd E Golde, Leonard Petrucelli

**Affiliations:** 1 Department of Neuroscience, Mayo Clinic, Jacksonville, FL 32224, USA

Over the last several decades there has been great progress with respect to understanding the triggers of most neurodegenerative diseases. For example, genetics studies have identified mutations in superoxide dismutase 1 (SOD1) gene that clearly cause amyotrophic lateral sclerosis. However ask a panel of experts how a mutation in SOD1 causes motor neurons to die and one will receive many different hypothetical answers. There is simply insufficient data to unequivocally support one hypothesis over another. With rare exceptions, this is true for almost all neurodegenerative diseases.

In this review series, we have invited seminal researchers in the field of neurodegeneration to critically review the pathways that potentially lead to neurodegeneration. These reviews include both disease specific reviews (e.g., the role of alpha-synuclein in driving neuronal death in Parkinson's disease) as well as more general reviews that focus on a pathway or a process that could lead to neurodegeneration (e.g., the role of autophagy in neurodegeneration).

As one of us describes in detail in the introductory review "The therapeutic importance of understanding mechanisms of neuronal cell death in neurodegenerative diseases" the effort to understand the precise pathway that lead to neuronal death is not simply academic. It has major implications in developing new therapies that might actually modify the disease process. By providing a "state of the field", it is our hope that this review series will reinvigorate efforts to conduct research that will ultimately answer the question, "What kills neurons in neurodegenerative diseases?" In each of the reviews, we have asked the authors to not only provide a comprehensive review of the key observations that demonstrate the importance of a specific protein or pathway in neurodegeneration but also highlight the "critical next steps" needed to shed light on the many enigmatic aspects of neurodegeneration.

Over 20 review articles will appear in this series during 2009. These reviews, published in the open access form in *Molecular Neurodegeneration*, will allow broad public access. Given the flexibility of this publishing format and the wide range of subject matter that could be addressed, we encourage those wishing to contribute substantive reviews of material not currently covered to contact either of us to discuss a potential submission.

